# Controlling the Crystallinity and Morphology of Bismuth Selenide via Electrochemical Exfoliation for Tailored Reverse Saturable Absorption and Optical Limiting

**DOI:** 10.3390/nano15010052

**Published:** 2024-12-31

**Authors:** Hao Yan, Bingxue Li, Junjie Pan, Xuan Fang, Yongji Yu, Dengkui Wang, Dan Fang, Yanyan Zhan, Xiaohua Wang, Jinhua Li, Xiaohui Ma, Guangyong Jin

**Affiliations:** 1 State Key Laboratory of High Power Semiconductor Lasers, School of Physics, Changchun University of Science and Technology, Changchun 130022, China; yh0224@cust.edu.cn (H.Y.); 2021100125@mails.cust.edu.cn (B.L.); pjj18943122097@163.com (J.P.); yyjcust@163.com (Y.Y.); wccwss@foxmail.com (D.W.); fangdan19822011@163.com (D.F.); zhanyanyan5876@163.com (Y.Z.); biewang2001@126.com (X.W.); lijh@cust.edu.cn (J.L.); mxh@cust.edu.cn (X.M.); 2 Jilin Key Laboratory of Solid Laser Technology and Application, School of Science, Changchun University of Science and Technology, Changchun 130022, China; 3 Chongqing Research Institute, Changchun University of Science and Technology, No.618 Liangjiang Avenue, Longxing Town, Yubei District, Chongqing 401135, China

**Keywords:** Bi_2_Se_3_, electrochemistry, nonlinear optical absorption, reverse saturable absorption, OA Z-scan technique

## Abstract

As an emerging two-dimensional (2D) Group-VA material, bismuth selenide (Bi_2_Se_3_) exhibits favorable electrical and optical properties. Here, three distinct morphologies of Bi_2_Se_3_ were obtained from bulk Bi_2_Se_3_ through electrochemical intercalation exfoliation. And the morphologies of these nanostructures can be tuned by adjusting solvent polarity during exfoliation. Then, the nonlinear optical and absorption characteristics of the Bi_2_Se_3_ samples with different morphologies were investigated using open-aperture Z-scan technology. The results reveal that the particle structure of Bi_2_Se_3_ exhibits stronger reverse saturable absorption (RSA) than the sheet-like structure. This is attributed to the higher degree of oxidation and greater number of localized defect states in the particle structure than in the sheet-like structure. Electrons in these defect states can be excited to higher energy levels, thereby triggering excited-state and two-photon absorption, which strengthen RSA. Finally, with increasing the RSA, the optical limiting threshold of 2D Bi_2_Se_3_ can also be increased. This work expands the potential applications of 2D Bi_2_Se_3_ materials in the field of broadband nonlinear photonics.

## 1. Introduction

Among potential nonlinear optical (NLO) materials, two-dimensional (2D) NLO materials with appropriate response times, substantial nonlinear polarization, and high optical homogeneity, laser damage threshold, nonlinear absorption (NLA) coefficient [1,2,3,4], and transparency are used in a wide range of applications, including optical switches [5], optical limiting [6], mode-locked lasers [7], and optical sensor/human eye protection [8]. Reverse saturable absorption (RSA) is a NLO process in which the optical absorption of materials increases with the excitation intensity. Certain 2D materials with RSA characteristics have been used extensively in the fields of optical limiting, ultrafast optical switching, and optoelectronic detection [9]. In 2017, Varma et al. used the open-aperture (OA) z-scan technique to demonstrate that 2D TiS_2_ nanosheets have a strong optical limiting (OL) effect. They also used the photoacoustic z-scan technique to reveal that the OL effect of 2D Ti_2_S nanosheets is mostly caused by NLA rather than scattering effects [10]. However, the NLO performance of materials is closely related to their synthesis method; the same material (e.g., MoS_2_ [11,12], Ti_3_C_2_T_x_ [13,14], black phosphorus [15,16], or perovskites [17]) prepared by a different method can exhibit different or even opposing properties. Enhancing NLO performance necessitates the development of reproducible synthesis methods and the establishment of structure–property relationships. However, there has been a scarcity of research publications in this area to date.

Two-dimensional bismuth selenide (Bi_2_Se_3_) materials have attracted considerable attention from researchers because of their unique physical properties as topological insulators with high carrier mobility and a narrow bandgap [18]. Two-dimensional Bi_2_Se_3_ possesses a layered structure in which layers are weakly bonded through van der Waals interactions. Each layer is composed of Bi and Se atoms covalently bonded in the sequence Se^1^-Bi-Se^2^-Bi-Se^1^ [19]. The topological surface states of Bi_2_Se_3_ exhibit a linear Dirac dispersion and helical spin texture, resulting in physical phenomena, such as spin-polarized surface currents [20]. The surface bandgap of Bi_2_Se_3_ is modulated by thickness and the occupancy of empty surface states, which enables the control of its photoresponse. Bi_2_Se_3_ has been studied in the fields of visible-infrared detection [21,22], terahertz detection [23,24], optical spintronic devices [25], and nonlinear optics [26]. In particular, 2D Bi_2_Se_3_ with third-order NLO properties shows potential for use in optical limiting, optical switching, and mode-locking laser systems. In 2023, Haris et al. achieved passive Q-switching and mode-locking in erbium-doped fiber lasers using Bi_2_Se_3_ prepared by optical deposition as a saturable absorber [7]. Electrochemical exfoliation is an efficient and environmentally friendly method for preparing low-dimensional materials. In 2016, Adriano and co-workers obtained monolayer and few-layer Bi_2_Se_3_ from natural crystals through electrochemical exfoliation [27]. A large proportion of surface atoms is a distinctive feature of 2D materials, and so the band structure is susceptible to surface adsorbents and/or surface terminations; such surface terminations are anticipated to have a strong influence on the NLO absorption properties of 2D materials, especially ultrathin 2D materials [28,29,30]. Reports on two-dimensional Bi_2_Se_3_ in relevant fields are relatively scarce, with a particular lack of research on the structural control of two-dimensional Bi_2_Se_3_ and its nonlinear optical (NLO) properties.

In this work, we employ an electrochemical exfoliation method with conditions that are controlled using three different polar solvents and detergents to obtain low-dimensional Bi_2_Se_3_ crystals with three distinct morphologies [31,32,33]. The influence of the solvent on the morphology of Bi_2_Se_3_ crystals formed during the exfoliation process is studied using scanning electron microscopy (SEM), transmission electron microscopy (TEM), energy-dispersive X-ray spectroscopy (EDS), and X-ray photoelectron spectroscopy (XPS). The exfoliated Bi_2_Se_3_ is included in polymethylmethacrylate (PMMA) organic glasses and their NLO optical properties are investigated using the OA z-scan technique. The results indicate that all three types of Bi_2_Se_3_/PMMA glasses exhibit RSA characteristics. The RSA effect diminishes as the polarity of the delamination solvent increases. Bi_2_Se_3_-MeCN/PMMA has a relatively large NLA coefficient of 1.8 ± 0.05 cm/GW and an optical limiting (OL) threshold of 3 J/cm^2^. Our results indicate that Bi_2_Se_3_ nanosheet OL materials have great potential for protecting optical components and human eyes from damage by lasers.

## 2. Materials and Methods

### 2.1. Electrochemical Exfoliation of Bi_2_Se_3_

**Figure 1:**
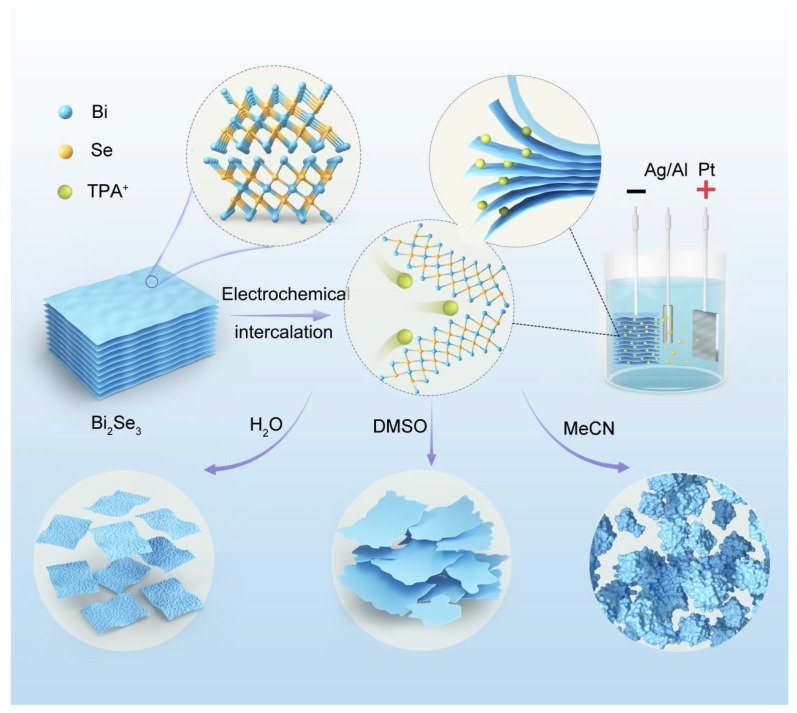
Illustration of the electrochemical exfoliation of Bi_2_Se_3_.

The exfoliation process of Bi_2_Se_3_ is illustrated in Figure 1. A three-electrode electrochemical system was constructed, utilizing a small bulk Bi_2_Se_3_ (20 mm × 20 mm) as the working electrode, platinum foil as the counter electrode, and Ag/AgCl as the reference electrode for intercalation reactions. The small bulk was connected to an alligator clip via a copper strip. During the connection, the small bulk and copper strip were wrapped with sealing film to prevent copper contamination of the electrolyte solution while ensuring the flow of current and ions, exposing only the bulk to the solution. In the electrochemical exfoliation method, the intercalation of solvent molecules is a crucial factor affecting the morphology and composition of the exfoliated product, determining the mobility of active ions and the strength of charge capacity during the electrochemical exfoliation process. Here, we investigated the influence of three different polar solvents on the intercalation morphology and structure, selecting tetrapropylammonium bromide (TPA+) as the intercalation cation, dissolved in an electrolyte containing water, sodium sulfate (Na_2_SO_4_), dimethyl sulfoxide (DMSO, 3.2 mg mL^−1^), and acetonitrile (MeCN, 5 mg mL^−1^), with precise control over the content of the three solvents to ensure a stable and efficient exfoliation process. A bias of −5 V was applied for 15 min to induce exfoliation. After exfoliation, the electrolyte containing Bi_2_Se_3_ was centrifuged five times for 5 min at 5000 Revolutions Per Minute. During centrifugation, the Bi_2_Se_3_ products were washed with deionized water, DMSO, or MeCN to remove electrolytes. Finally, the deposits were dried at 80 °C for 24 h. The sample that was fabricated using the aqueous Na_2_SO_4_ electrolyte solution and washed with deionized water is named Bi_2_Se_3_-H_2_O. The sample that was fabricated using the DMSO electrolyte solution and washed with DMSO is named Bi_2_Se_3_-DMSO. The sample that was fabricated using the MeCN electrolyte solution and washed with MeCN is called Bi_2_Se_3_-MeCN.

### 2.2. Characterization

Bismuth (99.999%), selenium (99.999%) were obtained from Beike 2D materials Co., Ltd., Suzhou, China. The tetrapropylammonium bromide, dimethyl sulfoxide, acetonitrile, and sodium sulfate H_2_O were purchased from Shanghai Aladdin Biochemical Technology Co., Ltd., Shanghai, China. The electrochemical workstation was purchased from the Changchun Institute of Applied Chemistry Academia of Sciences. XPS (K-Alpha+, Thermo Fisher Scientific, Waltham, MA, USA) was used to identify the chemical state of bismuth; the spectra were calibrated using the C 1s band at 284.8 eV. SEM was performed using (Hitachi FE-SEM S4800, Hitachi, Ltd., Tokyo, Japan) at 5 kV and (Helios Nanolab G3 UC, FEI, Ltd., Hillsboro, OR, USA) at 5 kV. Detailed structural information was collected using high-resolution bright-field TEM (Talos F200S, Thermo Fisher Scientific, Waltham, MA, USA) and the composition was analyzed by EDS. The Z-scan system (z-scan) used in this work to determine the nonlinear optical (NLO) properties was from Changchun New Industries Optoelectronics Technology Co., Ltd., Changchun, China.

## 3. Results and Discussion

**Figure 2:**
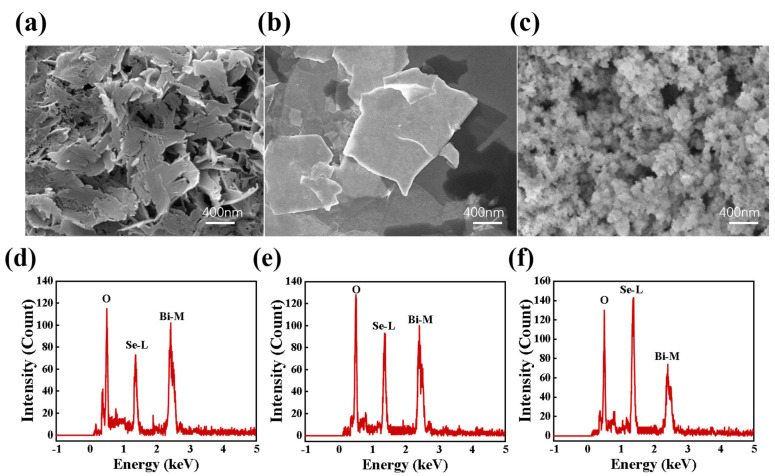
SEM images of (**a**) Bi_2_Se_3_-H_2_O, (**b**) Bi_2_Se_3_-DMSO, and (**c**) Bi_2_Se_3_-MeCN. EDS results of (**d**) Bi_2_Se_3_-H_2_O (**e**), Bi_2_Se_3_-DMSO, and (**f**) Bi_2_Se_3_-MeCN.

SEM images of the Bi_2_Se_3_ samples are shown in Figure 2a–c. Three types of morphologies of Bi_2_Se_3_ structures were obtained depending on the used electrolyte. Bi_2_Se_3_-H_2_O had an obvious thin-layer structure (Figure 2a), Bi_2_Se_3_-DMSO (Figure 2b) consisted of larger and thicker flake-like structures than those of Bi_2_Se_3_-H_2_O, and in Figure 2c it is shown that Bi_2_Se_3_-MeCN consists of stacked particles that are clusters of small nanosheets with a relatively large number of spherical nanoparticles that are about 30–50 nm in diameter. For a detailed enlarged view, please refer to Figure S1 in the Supplementary Materials. The EDS results show that Bi_2_Se_3_-H_2_O, Bi_2_Se_3_-DMSO, and Bi_2_Se_3_-MeCN are composed of O, Bi, and Se (Figure 2d–f). However, the O content is not the same in the three samples, Bi_2_Se_3_-H_2_O being the one with the lowest O content and Bi_2_Se_3_-MeCN with the highest. This observation illustrates the considerable influence of the solvent on the O content of Bi_2_Se_3_ materials produced by electrochemical exfoliation. The XRD and AFM characterization data can be found in the Supplementary Materials (Figures S2 and S3).

**Figure 3:**
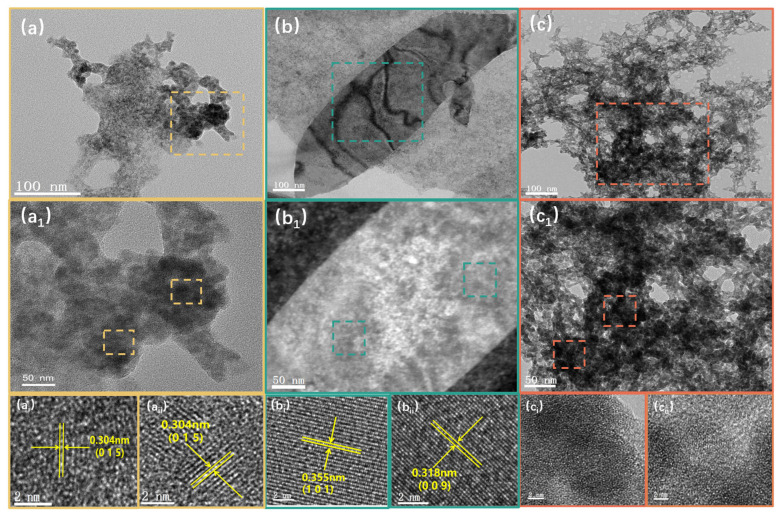
TEM images of Bi_2_Se_3_-H_2_O (**a**,**a_1_**,**a_i_**,**a_ii_**) at different magnifications; TEM images of Bi_2_Se_3_-DMSO (**b**,**b_1_**,**b_i_**,**b_ii_**) at different magnifications; TEM images of Bi_2_Se_3_-MeCN (**c**,**c_1_**,**c_i_**,**c_ii_**) at different magnifications.

The crystallinity was characterized using transmission electron microscopy (TEM). As shown in Figure 3a,b, the Bi_2_Se_3_-H_2_O sample predominantly exhibits small flake dimensions, while the Bi_2_Se_3_-DMSO sample demonstrates relatively larger lateral dimensions. The Bi_2_Se_3_-MeCN sample consists of aggregated small particles (Figure 3c). Upon examination of the magnified images of Bi_2_Se_3_-H_2_O and Bi_2_Se_3_-DMSO (Figure 3(a_i_,a_ii_,b_i_,b_ii_)), the lattice spacing of Bi_2_Se_3_-H_2_O is 0.304 nm, corresponding to the distance of the (0 1 5) plane of the crystal, and it exhibits partial amorphization due to oxidation. The lattice spacings of Bi_2_Se_3_-DMSO are 0.355 nm and 0.318 nm, corresponding to the (1 0 1) and (0 0 9) crystal planes; high-resolution images show almost no amorphization due to oxidation. In contrast to the first two samples, the magnified TEM image of Bi_2_Se_3_-MeCN (Figure 3(c_i_,c_ii_)) reveals its amorphous state, lacking distinct lattice fringes.

During electrochemical exfoliation, the polarity of the electrolyte solution affects the diffusion of electrolyte ions between the Bi_2_Se_3_ layers. In a solvent with low polarity, the depth to which ions can diffuse is small. A higher dielectric constant is correlated with higher polarity and consequently a greater ability to stabilize charges. At room temperature, the dielectric constants of MeCN, DMSO, and deionized water are 36.7, 47, and 78.3, respectively. Therefore, the electrolytes possess the following order of polarity: MeCN < DMSO < deionized water. In a solution with low polarity, the depth to which ions can diffuse is small, which results in the formation of Bi_2_Se_3_-MeCN particles. In a more polar solution, the ions can diffuse more deeply into Bi_2_Se_3_, which resulted in Bi_2_Se_3_-H_2_O forming flakes. Additionally, oxygen is more readily adsorbed onto the surface of samples in solutions with low polarity, which explains why Bi_2_Se_3_-MeCN exhibited a higher degree of oxidation than the other samples. Oxidation can also lead to the disruption of the lamellar crystal structure, resulting in the formation of an amorphous structure. In solutions with high polarity, oxygen adsorption is suppressed, which is why only a small amount of oxidation occurred at the edges of the Bi_2_Se_3_-H_2_O flakes. Because the MeCN solution had the lowest polarity of the electrolytes, it allowed more oxygen to penetrate the exfoliated Bi_2_Se_3_-MeCN sample. Oxygen adsorption by the exfoliated samples leads to the formation of Bi-O-Bi bonds within the Bi_2_Se_3_ nanosheets. As the oxidation degree increases, additional Bi-O bonds form. Consequently, the lattice structure of Bi_2_Se_3_-MeCN is disrupted by oxidation, leading to the formation of amorphous Bi_2_Se_3_. In contrast, the DMSO solution had relatively neutral polarity, which resulted in the formation of thicker flake structures. When rinsed with deionized water, Bi_2_Se_3_-H_2_O was primarily oxidized at the flake edges, whereas the central region maintained its crystalline structure. This results in the formation of distinct crystalline and amorphous states. This lays an experimental foundation for the controlled formation of Bi_2_Se_3_ under non-oxidative atmosphere conditions.

**Figure 4:**
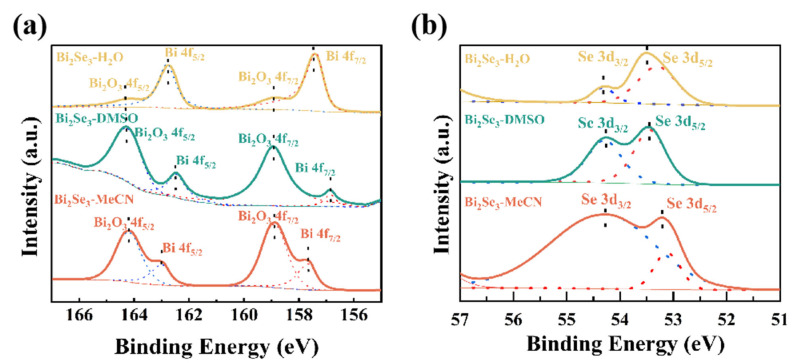
High-resolution (**a**) Bi 4f spectra and (**b**) Se 3d spectra of Bi_2_Se_3_-H_2_O, Bi_2_Se_3_-DMSO, and Bi_2_Se_3_-MeCN.

XPS was employed to investigate the composition and bonding energy of Bi_2_Se_3_. As shown in Figure 4a, the Bi 4f peak of Bi_2_Se_3_-H_2_O, Bi_2_Se_3_-DMSO, and Bi_2_Se_3_-MeCN was split into two peaks centered at 157 and 162.9 eV [34,35]. Because of oxidation during the exfoliation process, the Bi 4f spectra also contained peaks from Bi_2_O_3_ at 158.9 and 164 eV. The Bi_2_O_3_ peak intensity was the highest for Bi_2_Se_3_-MeCN and lowest for Bi_2_Se_3_-H_2_O. When oxygen molecules penetrate the Bi_2_Se_3_ layers, they initially form Bi-O-Bi bonds. As more oxygen molecules enter, some of the Bi-O-Bi bonds break to form Bi-O bonds, which become dominant. Because the MeCN solution had the lowest polarity, Bi_2_Se_3_-MeCN exhibited the highest degree of oxidation and lowest crystallinity. In contrast, Bi_2_Se_3_-H_2_O only adsorbed oxygen at its flake edges, so the intensity of the Bi_2_O_3_ peak of Bi_2_Se_3_-H_2_O was weak. Figure 4b shows that the samples exhibited Se 3d peaks centered at 53.4 and 54.3 eV. The former peak is related to the divalent Se-(Se^2−^) of Se 3d_5/2_ in the Bi_2_Se_3_ lattice, whereas the latter is ascribed to Se 3d_3/2_.

**Figure 5:**
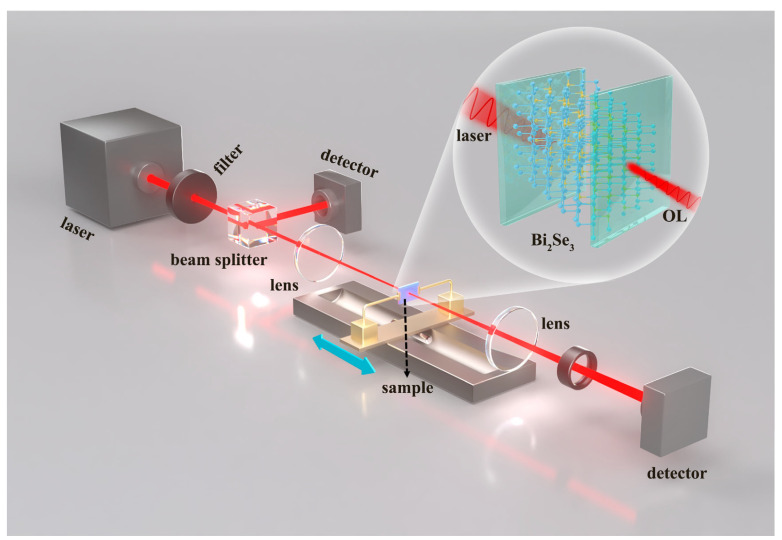
Schematic illustration of the open-aperture z-scan setup.

The traditional open-aperture (OA) Z-scan technique was employed to investigate the nonlinear optical (NLO) properties of the prepared Bi_2_Se_3_ samples. The samples were fixed on an electronic translation platform for z-scan measurements. The experimental arrangement is illustrated in Figure 5. The excitation laser was an Nd:YAG laser with a 6 ns pulse duration at 532 nm and repetition rate of 1 Hz. The output light first passed through the attenuator to form the incident light. The incident light was then divided into measurement and reference beams by a spectroscope. The reference beam was collected by detector 1. The measurement light was focused through a convex lens, passed through the sample, and was then received and recorded by detector 2. The electronic translation platform holding the sample was positioned along the optical path and moved from left to right. The energy of the control and transmitted beams of the sample was recorded at different locations.

**Figure 6:**
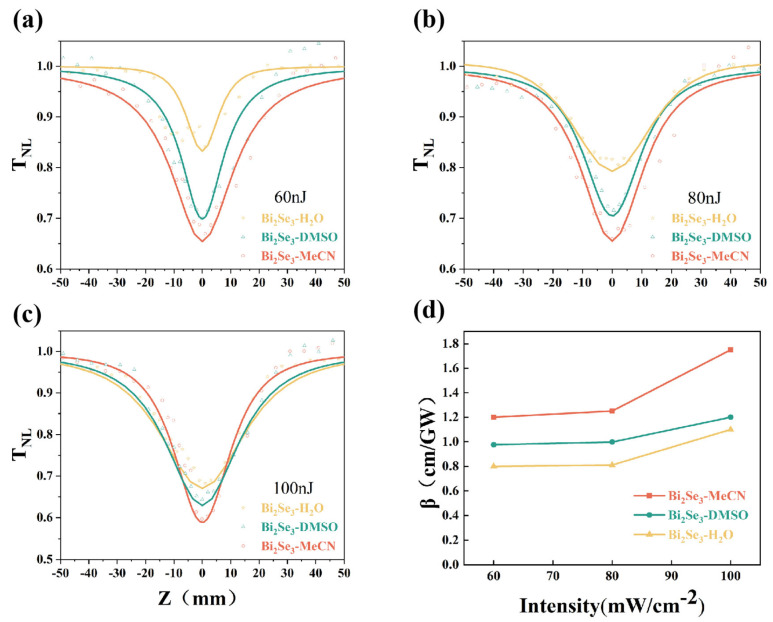
NLA results for Bi_2_Se_3_-H_2_O/PMMA, Bi_2_Se_3_-DMSO/PMMA, and Bi_2_Se_3_-MeCN/PMMA at (**a**) 60 μJ, (**b**) 80 μJ, and (**c**) 100 μJ. (**d**) Relationship between the excitation intensity and NLA coefficient (β) for the Bi_2_Se_3_/PMMA samples.

The effects of solvent polarity on the NLO properties of the Bi_2_Se_3_ samples was assessed by z-scan measurements. The three types of 2D Bi_2_Se_3_ prepared by electrochemical exfoliation were dispersed in PMMA to fabricate samples for z-scan testing; these samples are named Bi_2_Se_3_-H_2_O/PMMA, Bi_2_Se_3_-DMSO/PMMA, and Bi_2_Se_3_-MeCN/PMMA. OA z-scan experiments were conducted on the samples at different incident energies. Figure 6a–c show the normalized transmittance of the samples at energies of 60, 80, and 100 μJ, respectively. The physical images of Bi_2_Se_3_/PMMA organic glass are presented in Figure S4.

As shown in Figure 6a, at 60 μJ, the curves of the three samples were symmetric around a Rayleigh length (*Z*) of 0. The samples produced a downward peak shape at 532 nm, which was consistent with RSA. When each sample approached *Z* = 0, its transmittance was at its minimum. At an energy of 60 μJ, the transmittance values of Bi_2_Se_3_-H_2_O/PMMA, Bi_2_Se_3_-DMSO/PMMA, and Bi_2_Se_3_-MeCN/PMMA were 0.83, 0.69, and 0.66, respectively. At the same energy, the peak value of the RSA curve increased with the decreasing polarity of the electrolyte. In the case of RSA, as the intensity of the incident light increased, the amount of light transmitted through Bi_2_Se_3_-MeCN/PMMA decreased, that is, a larger proportion of the incident light was absorbed by the sample. The same situation is depicted in Figure 6b,c. At an incident energy of 80 μJ, the transmittance values of Bi_2_Se_3_-H_2_O/PMMA, Bi_2_Se_3_-DMSO/PMMA, and Bi_2_Se_3_-MeCN/PMMA were 0.8, 0.7, and 0.65, respectively. At 100 μJ, the transmittance values of Bi_2_Se_3_-H_2_O/PMMA, Bi_2_Se_3_-DMSO/PMMA, and Bi_2_Se_3_-MeCN/PMMA were 0.67, 0.62, and 0.59, respectively. Under the three incident light intensities, Bi_2_Se_3_-H_2_O/PMMA displayed the highest transmittance, Bi_2_Se_3_-DMSO/PMMA was in the middle, and Bi_2_Se_3_-MeCN/PMMA exhibited the lowest transmittance. The lowest transmittance observed for these samples was 0.59. These results indicate that a higher degree of an amorphous structure leads to stronger RSA, i.e., Bi_2_Se_3_-MeCN/PMMA shows the greatest RSA of the samples.

Based on the abovementioned RSA characteristics, the NLA coefficients of the plexiglass samples were further analyzed. The normalized transmittance (*T*) can be determined from OA z-scan measurements using the equation *T* = (1 − α*l*)/(1 − α_0_*l*), where α and α_0_ are the total absorption coefficient and linear absorption coefficient, respectively, and l is the thickness of the sample, which is 1 mm. α can be calculated using Equation (1), where *I*(*z*) is the power density that varies with the sample movement, *I_s_* is the saturable absorption intensity, and β is the NLA coefficient. Equation (2) is used to calculate for *I*(*z*), where *I*_0_ is the peak power density and *Z*_0_ is the coordinate of the sample during the OA z-scan measurements. Eventually, we obtain a deformation formula for *T*, as shown in Equation (3):

(1) $$\alpha =\frac{\alpha_{0}}{1+I(Z)/I_{S}}++\beta I(z),$$

(2) $$I(z)=\frac{I_{0}}{1+Z^{2}/Z_{0}^{2}},$$

(3) $$T=[1-\frac{\alpha_{0}I_{S}l}{I_{S}+I_{0}/(1+Z^{2}/Z_{0}^{2})}-\frac{\beta I_{0}l}{1+Z^{2}/Z_{0}^{2}}]/(1-\alpha_{0}I).$$

We obtained β values of the three types of Bi_2_Se_3_ acrylics at different excitation energies and 532 nm, as shown by the solid lines in Figure 6c. At 60 μJ, β values for Bi_2_Se_3_-H_2_O/PMMA, Bi_2_Se_3_-DMSO/PMMA, and Bi_2_Se_3_-MeCN/PMMA were 0.8 ± 0.05, 1.0 ± 0.05, and 1.2 ± 0.05 cm/GW, respectively. At 80 μJ, β values for Bi_2_Se_3_-H_2_O/PMMA, Bi_2_Se_3_-DMSO/PMMA, and Bi_2_Se_3_-MeCN/PMMA were 0.83 ± 0.05, 1.2 ± 0.05, and 1.24 ± 0.05 cm/GW, respectively. At 100 μJ, β values for Bi_2_Se_3_-H_2_O/PMMA, Bi_2_Se_3_-DMSO/PMMA, and Bi_2_Se_3_-MeCN/PMMA were 1.11 ± 0.05, 1.19 ± 0.05, and 1.8 ± 0.05 cm/GW, respectively. As the excitation energy increased, β also increased, so a higher energy corresponded to a stronger OL effect. Moreover, under the same excitation energy, Bi_2_Se_3_-H_2_O/PMMA showed the highest β among the three types of acrylics, followed by Bi_2_Se_3_-DMSO/PMMA and then Bi_2_Se_3_-MeCN/PMMA. Therefore, the crystalline state of Bi_2_Se_3_ determines its NLA properties. The mechanism behind this behavior can be explained as follows. Compared with Bi_2_Se_3_-H_2_O and Bi_2_Se_3_-DMSO, Bi_2_Se_3_-MeCN has more localized defect states because of its increased oxidation. The defect states assist electronic transitions from the valence band to the conduction band by absorbing photons. Simultaneously, electrons from the defect states can be excited to higher energy states, thus triggering excited-state absorption and two-photon absorption, leading to stronger RSA characteristics. Furthermore, Bi_2_Se_3_-MeCN has the highest degree of oxidation, followed by Bi_2_Se_3_-DMSO and then Bi_2_Se_3_-H_2_O. The likelihood of auxiliary transitions caused by defect states decreases from a high to low degree of oxidation, which also leads to a gradient change in β.

**Figure 7:**
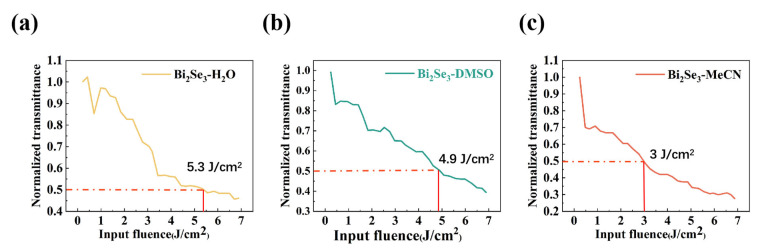
Relationship between the normalized transmittance and the intensity: (**a**) Bi_2_Se_3_-H_2_O; (**b**) Bi_2_Se_3_-DMSO; (**c**) Bi_2_Se_3_-MeCN.

The crystallinity of Bi_2_Se_3_ influences its OL effect. Compared with Bi_2_Se_3_-H_2_O and Bi_2_Se_3_-DMSO, Bi_2_Se_3_-MeCN has more localized defect states because of its higher degree of oxidation. This facilitates the transition of electrons from the valence band to the conduction band by absorbing photons. Electrons can transfer from the valence band to the defect states, and at the same time, electrons in the defect states can also be excited to higher energy states, thereby triggering excited-state absorption and two-photon absorption, which are RSA characteristics [36,37,38]. As shown in Figure 7a–c, the nonlinear threshold of 50% transmittance at 532 nm for the three types of Bi_2_Se_3_ nanosheets are 5.3, 4.9, and 3 J/cm^2^, respectively. The degree of oxidation of Bi_2_Se_3_-DMSO is lower than that of Bi_2_Se_3_-MeCN, which decreases the likelihood of auxiliary transitions caused by defect states.

## 4. Conclusions

Electrochemical exfoliation was used to prepare 2D Bi_2_Se_3_ in a controllable manner. Altering the electrolyte and centrifugation wash solution affected the crystallinity of the products. The microstructure and spectral characteristics of the Bi_2_Se_3_ samples were analyzed. The oxidation mechanism of the products prepared under different conditions was examined. The influence of the crystallinity of Bi_2_Se_3_ on its NLO properties was studied. The results indicate that the NLA performance of Bi_2_Se_3_ can be tuned by adjusting its crystallinity. PMMA films of Bi_2_Se_3_-H_2_O, Bi_2_Se_3_-DMSO, and Bi_2_Se_3_-MeCN exhibit RSA characteristics. Bi_2_Se_3_-MeCN, which had a higher amorphous content than the other samples, showed the strongest RSA effect. Conversely, Bi_2_Se_3_-H_2_O, which had the highest crystallinity of the samples, exhibited the weakest RSA effect. This is because the RSA effect was primarily determined by the density of local defect states in the samples. Bi_2_Se_3_ can be considered an attractive NLA material. Bi_2_Se_3_ with a high amorphous content represents a promising platform to realize optical devices, such as optical limiters and shutters.

Further experimental details can be found in the Supplementary Materials, including atomic force microscopy data, X-ray diffraction patterns, scanning electron microscope enlargements, and physical images of Bi_2_Se_3_/PMMA organic glass.

## Data Availability

Data are contained within the article and Supplementary Materials.

## Supplementary Materials

The following supporting information can be downloaded at https://www.mdpi.com/article/10.3390/nano15010052/s1, Figure S1: SEM images of (a) Bi_2_Se_3_-H_2_O, (b) Bi_2_Se_3_-DMSO, and (c) Bi_2_Se_3_-MeCN. Figure S2: XRD results of Bi_2_Se_3_-H_2_O Bi_2_Se_3_-DMSO and Bi_2_Se_3_-MeCN. Figure S3: AFM results of (a) Bi_2_Se_3_-H_2_O (b) Bi_2_Se_3_-DMSO and (c) Bi_2_Se_3_-MeCN. Figure S4: Bi_2_Se_3_/PMMA Plexiglass finished product.
