# Supplementary material for: Controlling the Crystallinity and Morphology of Bismuth Selenide via Electrochemical Exfoliation for Tailored Reverse Saturable Absorption and Optical Limiting
Source: Nanomaterials (Basel). 2024 Dec 31;15(1):52. doi: 10.3390/nano15010052 (PMC11723395; doi:10.3390/nano15010052)
Supplement: Supplementary file 1 [file nanomaterials-15-00052-s001.zip › nanomaterials-3296776-supplementary.pdf]

supplementary material

Controlling the Crystallinity and Morphology of Bismuth Selenide via Electrochemical Exfoliation for Tailored Reverse Saturable Absorption and Optical Limiting

(nanomaterials-3296776)

1. Experimental section

The exfoliation process of Bi_2Se_3 is illustrated in Figure 1. A three-electrode electrochemical system was constructed, utilizing a small bulk Bi_2Se_3 (20mm×20mm) as the working electrode, platinum foil as the counter electrode, and Ag/AgCl as the reference electrode for intercalation reactions. The small bulk was connected to an alligator clip via a copper strip. During the connection, the small bulk and copper strip were wrapped with sealing film to prevent copper contamination of the electrolyte solution while ensuring the flow of current and ions, exposing only the bulk to the solution. In the electrochemical exfoliation method, the intercalation of solvent molecules is a crucial factor affecting the morphology and composition of the exfoliated product, determining the mobility of active ions and the strength of charge capacity during the electrochemical exfoliation process. Here, we investigated the influence of three different polar solvents on the intercalation morphology and structure, selecting tetrapropylammonium bromide (TPA+) as the intercalation cation, dissolved in an electrolyte containing water, sodium sulfate (Na_2SO_4), dimethyl sulfoxide (DMSO, 3.2 $mg{\cdot}mL^{-1}$), and acetonitrile (MeCN, 5 $mg{\cdot}mL^{-1}$), with precise control over the content of the three solvents to ensure a stable and efficient exfoliation process. A bias voltage of -5 V was applied to the working electrode for 15 minutes to induce exfoliation. After exfoliation, the Bi_2Se_3-containing electrolyte was centrifuged at 5000 rpm for five cycles, each lasting 5 minutes. During the centrifugation process, the Bi_2Se_3 product was washed with deionized water, DMSO, or MeCN to remove the electrolyte. Finally,

the sediment was dried at 80 °C for 24 hours. Samples prepared with Na_2SO_4 aqueous solution and washed with deionized water were named Bi_2Se_3-H_2O, samples prepared with DMSO electrolyte solution and washed with DMSO were named Bi_2Se_3-DMSO, and samples prepared with MeCN electrolyte and washed with MeCN were termed Bi_2Se_3-MeCN.

Materials. Bismuth (99.999%), selenium (99.999%) were obtained from beike 2D materials Co., Ltd. The tetrapropylammonium bromide, Dimethyl sulfoxide, Acetonitrile, Sodium sulfate H_2O were purchased from Shanghai Aladdin Biochemical Technology Co., Ltd.The electrochemical workstation was purchased from Changchun Institute of Applied Chemistry Academia of Sciences.XPS (K-Alpha+, Thermo Fisher Scientific) was used to identify the chemical state of bismuth; the spectra were calibrated using the C 1s band at 284.8 eV. SEM was performed using a Hitachi FE-SEM S4800 at 5 kV and Helios Nanolab G3 UC at 5 kV. Detailed structural information was collected using high-resolution bright-field TEM (Talos F200S) and composition was analyzed by EDS. Z-scan system (z-scan) used in this work to determine nonlinear optical (NLO) properties from Changchun New Industries Optoelectronics Technology Co., Ltd.

2. Material characterizations

The samples were scanned by Hitachi FE-SEM S4800 and Helios Nanolab G3 UC at 5 kV and 5 kV respectively, and the details of the deposited particles and their structures in FigS1(c) were seen through the locally enlarged images. It can be seen from the enlarged image that the size of the spherical nanoparticles in Figure.S1 (c) is between30-50nm.

(a) **(b)** **(c)**

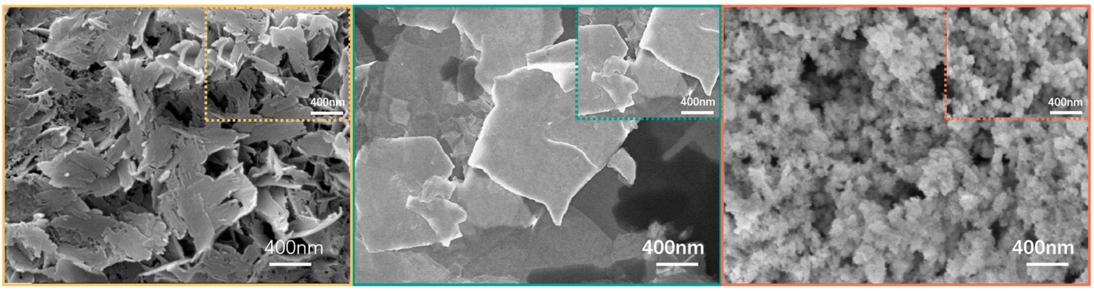

Figure. S1 SEM images of (a) Bi_2Se_3-H_2O, (b) Bi_2Se_3-DMSO, and (c) Bi_2Se_3-MeCN.

As shown in Figure S2. The diffraction peaks of three kinds of prepared B Bi_2Se_3 samples were obtained, and the corresponding position of peak (006) in the figure was 18.5°, which was consistent with the standard data. The results showed that the (006) peak half-peak width of Bi_2Se_3-H_2O was wider, the (006) peak half-peak width of Bi_2Se_3-DMSO was narrower, and the (006) peak half-peak width of Bi_2Se_3-MeCN was the widest. The results showed that among the three kinds of Bi_2Se_3 samples prepared, Bi_2Se_3-DMSO had the best crystallinity, followed by Bi_2Se_3-H_2O, and Bi_2Se_3-MeCN had the lowest crystallinity

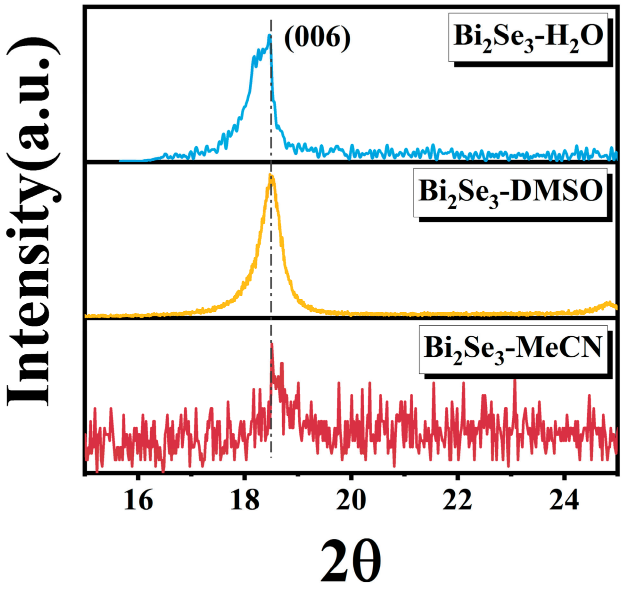

Figure. S2 XRD results of Bi_2Se_3-H_2O Bi_2Se_3-DMSO and Bi_2Se_3-MeCN.

This part has been revised according to your suggestion. We have supplemented the atomic force microscopy (AFM) test results for three types of low-dimensional nanomaterials, as shown in Figures S3(a), 4(b), and 4(c). The nanosheets, with lateral

dimensions ranging from 0.2 to 1 micrometer, exhibit a thickness of approximately 90 to 300 nanometers. Considering the agglomeration effect of the nanosheets during sample preparation, the actual thickness is theoretically somewhat less.

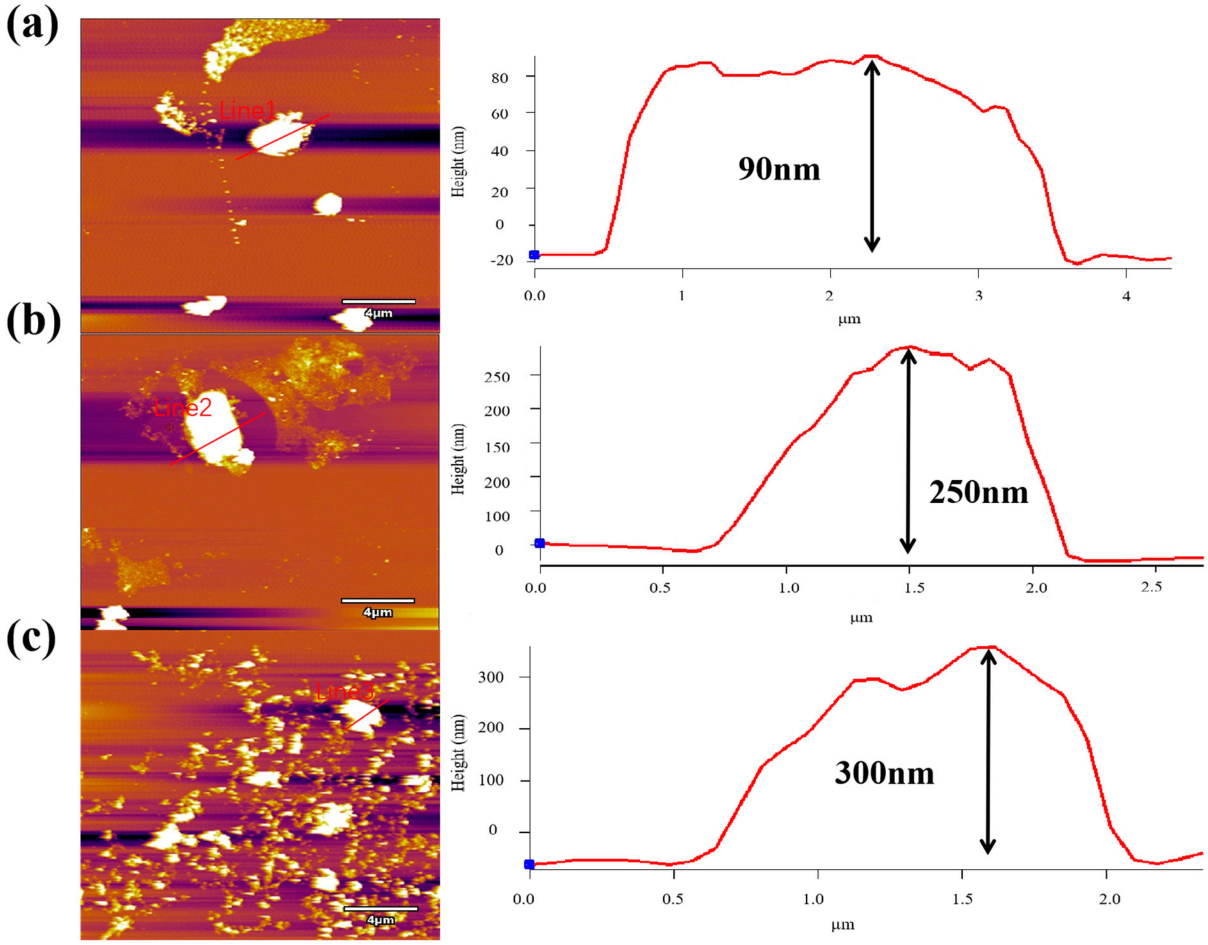

Figure. S3 AFM results of (a)Bi_2Se_3-H_2O (b) Bi_2Se_3-DMSO and (c) Bi_2Se_3-MeCN.

we provide the specific ratios and parameters for PMMA and Bi_2Se_3 as follows: Using an electronic balance, we weighed out 0.04 g of 2,2-azobisisobutyronitrile (AIBN), 10.35 g of methyl methacrylate (MMA), and 0.002 g of Bi_2Se_3-H_2O powder. Figure 6 shows the resulting Bi_2Se_3-H_2O/PMMA organic glass with good light transmittance, indicating that the Bi_2Se_3-H_2O powder is uniformly dispersed within the organic glass, which appears as a light gray color.

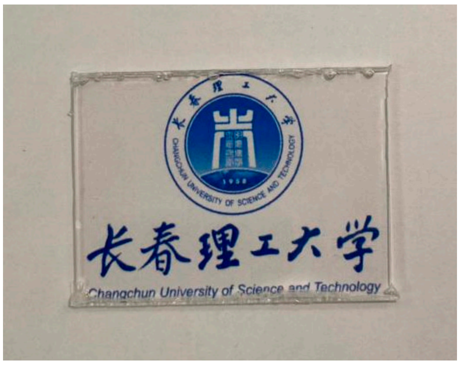

Figure.S4 Bi_2Se_3/PMMA Plexiglass finished product
